# Exposure to pesticides in Chile and its relationship with carcinogenic potential: a review

**DOI:** 10.3389/fpubh.2025.1531751

**Published:** 2025-04-01

**Authors:** María Teresa Muñoz-Quezada, Verónica Iglesias, Liliana Zúñiga-Venegas, Floria Pancetti, Claudia Foerster, Natalia Landeros, Boris Lucero, Daniel Schwantes, Sandra Cortés

**Affiliations:** ^1^School of Public Health, Faculty of Medicine, University of Chile, Santiago, Chile; ^2^Centro para la Prevención y el Control del Cáncer (CECAN), Santiago, Chile; ^3^Centro de Investigación de Estudios Avanzados del Maule, Vicerrectoría de Investigación y Postgrado. Universidad Católica del Maule, Talca, Chile; ^4^Laboratorio de Investigaciones Biomédicas, Facultad de Medicina, Universidad Católica del Maule, Talca, Chile; ^5^Laboratorio de Neurotoxicología Ambiental, Departamento de Ciencias Biomédicas, Facultad de Medicina, Universidad Católica del Norte, Coquimbo, Chile; ^6^Centro de Investigación y Desarrollo Tecnológico en Algas y otros Recursos Biológicos (CIDTA), Universidad Católica del Norte, Coquimbo, Chile; ^7^Institute of Agri-food, Animal and Environmental Sciences (ICA3), Universidad de O' Higgins, San Fernando, Chile; ^8^Unidad de Innovación en Prevención y Oncología de Precisión, Centro Oncológico, Facultad de Medicina, Universidad Católica del Maule, Talca, Chile; ^9^In Vivo Tumor Biology Research Facility, Centro Oncológico, Facultad de Medicina, Universidad Católica del Maule, Talca, Chile; ^10^The Neuropsychology and Cognitive Neurosciences Research Center CINPSI Neurocog, Faculty of Health Sciences, Universidad Católica del Maule, Talca, Chile; ^11^Escuela de Salud Pública, Facultad de Medicina, Pontificia Universidad Católica de Chile, Santiago, Chile; ^12^Departamento de Ciencias Vegetales, Facultad de Agronomía y Sistemas Naturales, Pontificia Universidad Católica de Chile, Santiago, Chile; ^13^Departamento de Química Física, Facultad de Química y de Farmacia, Pontificia Universidad Católica de Chile, Santiago, Chile; ^14^Advanced Center for Chronic Diseases (ACCDiS), Centro de Desarrollo Urbano Sustentable (CEDEUS), Santiago, Chile

**Keywords:** pesticide exposure, carcinogenic effects, environmental contamination, health risk assessment, genotoxicity

## Abstract

**Background:**

The widespread application of pesticides in agriculture and the consequent heightened human exposure to these potentially harmful substances present considerable environmental and health risks. The potential link to cancer is particularly concerning, underscoring the urgent need for more sustainable and health-conscious agricultural practices. Pesticides are pervasive global contaminants, with exposure occurring through various routes. Improper use is associated with genotoxicity, neurobehavioral problems, thyroid dysfunction, reproductive issues, and cancer, among other deleterious damages. While pesticide exposure is evident in Chile, a direct link to cancer remains uncertain.

**Objective:**

To examine the scientific evidence on pesticides exposure in the environment and human populations, and its relationship with cancer in Chilean territory.

**Methods:**

The search for original articles was performed in international peer-reviewed scientific databases, including Scopus, Web of Science (WoS), and PubMed Advanced Search Builder. Following the PRISMA extension for review guidelines, the search included studies on environmental pesticide exposure, human biomarker assessments, experimental investigations, and the potential pesticide-cancer associations in Chile. Foreign studies, systematic reviews, and meta-analyses were excluded.

**Results:**

Among the 83 qualifying studies conducted between 1996 and 2024, elevated pesticide exposure risks were documented, with 71% indicating high concentrations that may pose health risk. Additionally, 20% of studies reported significant chlorinated compounds, including organochlorines (OCs) and polychlorinated biphenyls (PCBs), while 79.5% identified carcinogenic to humans classified by the International Agency for Research on Cancer. The review emphasizes the urgent need to update pesticide-related regulations in Chile, including implementing bans or restrictions on carcinogenic pesticides and establishing stricter standards for allowable pesticide residue levels in food.

**Conclusion:**

Long-term epidemiological studies are essential to establishing concrete links between pesticide exposure and specific types of cancer. Additionally, investigating epigenetic markers associated with pesticide exposure, especially in occupational settings, is critical. Furthermore, allocating resources and prioritizing further research is fundamental, particularly in regions with substantial agricultural exposure.

## Introduction

Modern synthetic pesticides are manufactured chemical substances that constitute one of the primary contaminants in the agricultural environment, affecting natural ecosystems, populated areas, and cities worldwide (1). In the natural environment, pesticide residues are actually the result of chemical, physical, and biochemical degradation. The extent of their degradation depends on the chemical stability of the pesticide molecule and its physicochemical properties, which directly influence their interactions with various environmental compartments, such as soil, air, surface water, or groundwater (2–4). Consequently, the environmental persistence of these substances is influenced by a complex interplay between pesticide characteristics and environmental factors. This interplay determines their longevity and impacts in various ecological settings (4), ultimately leading to human exposure and the development of acute and chronic diseases.

Human population exposure to pesticides occurs in both occupational and residential settings through the consumption of food, water, and the inhalation of contaminated air. The main routes of exposure are dermal, oral, ocular, and respiratory (1). These compounds circulate through the bloodstream and are eliminated via urine, sweat, and exhaled air, allowing their measurement through biomarkers (5).

Without adequate personal protective measures, training, or proper information about pesticide applications near homes, schools, or workplaces, pesticides can lead to acute intoxications with symptoms such as skin irritation, nausea, dizziness, headaches, muscle weakness, seizures, coma, cardiac and respiratory arrest, and even death (6, 7) as well as chronic conditions including neurological disorders, behavioral alterations, sleep disturbances, congenital malformations, musculoskeletal abnormalities, endocrine disruptions, genotoxicity, kidney damage, and cancer, among others (8–13).

Agricultural activities, extensively studied in this context, involve both occupational and environmental exposure to pesticide. A review conducted in Latin America (13) revealed that the most frequent exposures are to organophosphate pesticides, herbicides, and pyrethroids, primarily affecting agricultural workers and populations living or studying near agricultural areas, including women and children. Among the main health effects evaluated in the region are neurobehavioral problems, thyroid function disorders, reproductive effects, and cancer.

While genetic alterations (genotoxicity) associated with pesticides exposure are well known and characterized, the epigenetic alterations of DNA responsible for the development of these pathologies, including carcinogenesis, have not yet been thoroughly investigated. Among these epigenetic alterations, DNA methylation, histone modifications, and microRNA deregulation have been the most studied in chronic pesticide exposure (14, 15).

The International Agency for Research on Cancer (IARC) classifies chemical substances based on their potential carcinogenicity to humans. Expert committees analyze global epidemiological, toxicological, and laboratory evidence to assign substances to one of four categories, depending on the strength of the available evidence. Substances classified as *Group 1* are confirmed to be carcinogenic to humans, while those in *Group 2A* are considered probably carcinogenic, and *Group 2B* possibly carcinogenic. When evidence is insufficient to determine carcinogenicity, substances are placed in *Group 3* (not classifiable as to their carcinogenicity in humans). Regarding certain pesticides, the IARC evaluates their potential link to cancer and categorizes them within this framework, considering the weight of scientific evidence available (16) (Table 1).

**Table 1:** Classification of chemical substances suspected of causing cancer according to the International Agency for Research on Cancer (16).

Group	Level of evidence	Examples*
1. Carcinogenic to humans	There is sufficient evidence in humans and animals to establish a causal relationship.	Lindane and pentachlorophenol
2A. Probably carcinogenic to humans	There is limited evidence in humans, but sufficient in animals to suggest possible carcinogenicity.	Non-arsenic insecticides used in occupational exposures include captafol, diazinon, dichlorodiphenyltrichloroethane (DDT), polychlorinated biphenyls (PCBs), dieldrin, aldrin, glyphosate, and malathion.
2B. Possibly carcinogenic to humans	There is limited evidence in humans and animals, but it is insufficient to establish a clear relationship of carcinogenicity.	1,3-dichloropropene, dichlorvos, herbicide 2,4-D (dichlorophenoxyacetic acid), HCHs (hexachlorocyclohexanes), HCB (hexachlorobenzene), diethanolamine, chlorothalonil, nitrobenzene, parathion, pyridine, diethanolamine and diphenylamine.
3. Carcinogenicity not classifiable	There is no adequate evidence in humans and animals to determine its carcinogenic capacity.	
4. Probably not carcinogenic	The available evidence does not suggest that the substance is carcinogenic in humans or animals.	

* PCBs, PAHs, HCB, and PBDEs are not pesticides, but persistent organic pollutants that enter the environment through similar pathways. Their inclusion in the table aims to provide a more comprehensive view of toxic chemical exposure in agricultural systems, as they can interact with pesticides and increase risks to human health and the environment.

Intensive pesticide application practices in Chile are implemented to maintain high productivity levels (17, 18). According to a national review, it has been demonstrated that exposure levels in both the general population and occupational settings are higher than those reported in international studies conducted on agricultural workers and the pediatric population. The most frequently observed effects are related to neurotoxicity, genotoxicity, and reproductive health (19, 20), with few studies examining the association with cancer.

In Chile, a series of agricultural pesticides have been banned between 1982 and 2022. However, the issue with this list of 34 active pesticide ingredients is that some are based solely on specific brand names containing the active compound, while others brands with similar active compounds, such as chlorpyrifos-methyl, were only recently banned in December 2022 or are still available in the market (21).

Pesticides containing active ingredients classified by the IARC as probably (2A) or possibly (2B) carcinogenic to human, including diazinon, malathion, 2,4-D, and glyphosate, continue to be sold in Chile, according to the most recent sales data (22). Similarly, organic compounds such as diethanolamine and diphenylamine, commonly used alongside pesticides in agricultural practices (16), remain available in the market.

It is essential to mention that although Chile recently became the first country in Latin America to ban old glyphosate formulations containing the surfactant polyethoxylated tallow amine (21, 23), glyphosate itself remains one of the most used molecules.

In Chile, there has been no comprehensive narrative compilation of evidence regarding the presence of pesticides in the environment, population exposure levels, and their potential association with cancer. It is important to note that many of the pesticides used in Chile and other South American countries are prohibited or restricted in most European Union and OECD countries due to their documented adverse effects on health and the environment (24).

The classification of glyphosate as “probably carcinogenic to humans” by the IARC in 2015 has been part of an ongoing scientific and regulatory discussion. In contrast, the European Food Safety Authority (EFSA), following its assessment framework, concluded that glyphosate does not meet the criteria for classification as a carcinogen under EU regulations (25). These differing conclusions reflect the complexities of chemical risk assessment, which depend on variations in methodology, data sources, and interpretation of evidence. Regulatory assessments may prioritize different types of studies, and ensuring transparency and consistency in evaluation processes remains essential for informed decision-making and public confidence (26).

The United Nations has disclosed a substantial 119.4% surge in pesticide usage across South America from 1999 to 2020, documented in kilograms per hectare of land (24). The concept of “chemical colonialism” has been used to describe the disproportionate burden of pesticide use in South America, where weaker regulatory frameworks allow for the widespread application of highly hazardous pesticides that are often banned or restricted in Europe and the United States (27).

The reports underscore a significant prevalence of highly hazardous products, constituting 25% in Chile, 29% in Argentina, and a concerning 49% in Brazil. It is important to note that such a pattern of highly hazardous products is not observed in Europe or the United States, where most of the chemical industry is located (27).

This increase represents the highest growth rate observed during the study period, indicating a notable rise in the region’s pesticide dependency over the last two decades. Consequently, there arises an imperative need for a comprehensive examination of studies conducted in Chile that address pesticide exposure and its correlation with cancer in the national population.

This narrative review aimed to examine the scientific evidence on pesticides exposure in the environment and human populations, and its relationship with cancer in Chilean territory. This information is urgent for documenting exposure levels, especially to potentially carcinogenic pesticides in human populations, and advancing the estimation of the disease burden associated with these exposures in the Chilean population. These data would help protect public health, preserve the environment, and promote safer and more sustainable agricultural practices.

## Methods

**Figure 1:**
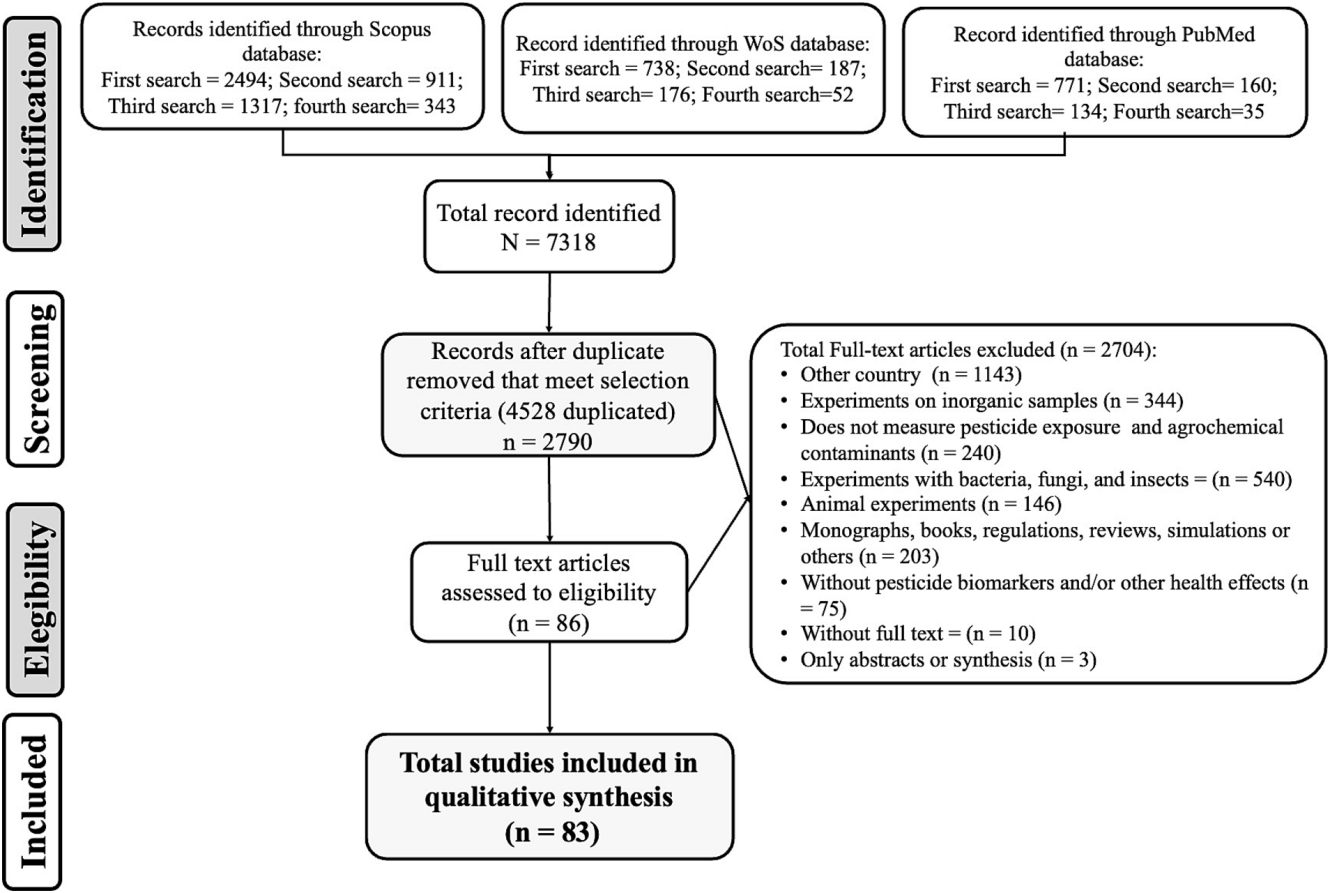
Summary diagram of identified texts (adapted from PRISMA-ScR) (28).

A bibliographic search was conducted based on the guidelines described in the PRISMA extension for reviews guidelines (PRISMA-ScR) (28), as shown in Figure 1.

The search for original articles was performed in international peer-reviewed scientific databases, including Scopus, Web of Science (WoS), and PubMed Advanced Search Builder. The “All Fields” option was selected to encompass the search terms comprehensively. All original manuscripts meeting the selection criteria were included up to December 30, 2024. A total of 12 searches were conducted (4 in Scopus, 4 in WoS, and 4 in PubMed) employing the following Boolean operators: “Pesticides and Chile not review”; “Pesticides and exposure and Chile not review”; “Pesticides and health and Chile not review”; “Pesticides and Cancer and Chile not review.”

The selection was limited to original publications in English, Spanish, or Portuguese. Studies conducted in Chile that involved environmental exposure to pesticides (vegetal, animal, water, soil, or air); studies examining exposure to pesticides with biomarkers in the Chilean population; Chilean experimental studies involving pesticides in human biological samples; and studies on pesticide exposure and cancer in the Chilean population were included. Excluded were studies involving foreign populations, systematic reviews, meta-analyses, monographs, or similar documents; experimental studies involving animals, plants, and inorganic samples; and studies lacking pesticide biomarkers or addressing other health issues.

The 12 searches were conducted by two researchers working simultaneously on manuscript selection. In the eligibility phase, we used Rayyan, a free website that facilitated the identification of duplicate manuscripts (29). The search results were compared among the seven researchers in team meetings until a 100% agreement was reached.

Subsequently, the analysis of the search results for national studies was synthesized in four sections: (a) description of studies related to the presence and quantified levels of pesticides in environmental matrices (water, sediments, plants, animals, soil, and air) in Chilean territory; (b) identification of studies assessing the presence of pesticide residues in fruits, vegetables, and other food consumed by the Chilean population; (c) studies evaluating biological pesticide exposure markers in human samples from the Chilean population; and (d) *in vitro* studies exploring pesticide exposure associated with carcinogenicity in human cell samples. The methodological quality of the studies was not evaluated.

The PECO (Population, Exposure, Comparison, Outcome) method (30) elements were used to summarize and describe pesticides exposure and related cancer studies. Additionally, a column was added to indicate whether some of the detected pesticides are classified as probably or possibly carcinogenic to humans (Group 1, 2A, and 2B) according to IARC (16) and which ones are allowed in Chile based on the review of SAG records and lists related to pesticide authorization and evaluation (31).

From Tables 2, 3 and Supplementary Table 1, a column titled “Concentration level as reported by each study reviewed (compared with international or national standards)” was included. The standards and criteria used to define whether pesticide concentrations were considered high or low were based on the Stockholm Convention, Codex Alimentarius Commission standards (32) for pesticide residues, regulations from the United States Environmental Protection Agency (33), and European Union (34) regulations on maximum residue levels (MRLs) in food products. For national standards, we used the regulations of the Chilean Ministry of Health (35) on maximum allowable pesticide residues in food and the records and lists from the Agricultural and Livestock Service (31) related to pesticide authorization and evaluation.

**Table 2:** Summary of studies related to pesticide exposure in ready-to-eat foods in Chilean territory (*n* = 11).

Author and year of publication	Macrozone	Design	Food	Pesticide(s) or other agrochemicals contaminants with higher concentration	Concentration level as reported by each study reviewed (compare with codex alimentarius or national standards)	Other pesticides or agrochemicals contaminants identified	Pesticides according to IARC classification (1, 2A and 2B)	Carcinogenic, probably or possibly carcinogenic to humans (Group 1, 2A or 2B) allowed in Chile
Concha-Meyer et al., 2019 (96)	South-Central	Longitudinal	Frozen fruits and vegetables	Iprodione and Spinosad A and D	High	Abamectin, chlorpyrifos, imidacloprid, and lambda cyhalothrin	No	No
Elgueta et al., 2017 (97)	Central and Metropolitan	Longitudinal	Vegetables (lettuce, chard, and spinach)	Methamidophos	High	cypermethrin, azoxystrobin, boscalide, cyfluthrin, chlorothalonil, chlorpyrifos, mancozeb, difenoconazole, esfenvalerate, imidacloprid, and labda-cyhalothrin	Chlorothalonil (2B)	Yes
Elgueta et al., 2019 (98)	Metropolitan	Longitudinal	Vegetables (lettuce, chard, and spinach)	Methamidophos	High	Carbendazim, cyfluthrin, chlorpyrifos, lambda-cyhalothrin, methamidophos	No	No
Elgueta et al., 2020 (99)	Metropolitan	Longitudinal	Vegetables (tomatoes and lettuce)	Methamidophos	High	Methomyl, difenoconazole, cyprodinil, and boscalid	No	No
Elgueta et al., 2021 (100)	Metropolitan	Longitudinal	Vegetables (tomatoes)	Methamidophos and chlorpyrifos	High	Methomyl	No	No
Fuentes et al., 2008 (101)	Metropolitan	Experimental	Olive oil and avocado	Diazinon	Low	Chlorpyrifos and methidathion	Diazinon (2A)	Yes
Fuentes et al., 2009 (102)	Central, Metropolitan, and South-Central	Experimental	Olive oil and avocado	Chlorpyrifos	Low	Diazinon, methidathion, and azinphos-methyl	Diazinon (2A)	Yes
Fuentes et al., 2010 (103)	Northern, Central, Metropolitan and South-Central	Longitudinal	Olive oil	Chlorpyrifos	Low	Diazinon, azinphos-methyl, and methidathion	Diazinon (2A)	Yes
Lapierre et al., 2019 (104)	Central, Metropolitan, South-Central, and South	Cross-sectional	Vegetables (lettuce, tomato), honey, milk, fruits (strawberries and raspberries)	Chlorothalonil, methamidophos, acetamiprid, tebuconazole	High	Iprodione, boscalid, pyraclostrobin, cyprodinil, bifenthrin, permethrin, and antimicrobials	Chlorothalonil (2B)	Yes
Muñoz-Quezada et al., 2014 (76)	South-Central	Cross-sectional	Fruits (apple, orange, pear) and vegetables (tomato and lettuce)	Diphenylamine and chlorpyrifos	Low	Thiabendazole, pyrimethanil, phosmet, tebuconazole, difenoconazole, myclobutanil, metaxyl, azinphos-methyl, iprodione, buprofezin, fenhexamid, lambda-cyhalothrin, triadimefon, carbaryl, dimethoate, cyprodinil, fludioxonil, and captan	Diazinon (2A), diphenylamine (2B)	Yes
Opazo-Navarrete et al., 2021 (105)	South	Cross-sectional	Vegetables (peas, corn, carrots, leeks, spinach, swiss chard, cilantro, and parsley)	2-phenylphenol, chlorpyrifos and mepanipyrim	High	Fenpropidine, cypermethrin, 2-phenylphenol, linuron, chlorpyrifos and mepanipyrim	No	No

**Table 3:** Summary of studies with exposure, effect, and susceptibility biomarkers of pesticides in biological samples of the Chilean population (*n* = 8).

Author and year of publication	Macrozone	Design	Sample and population	Metabolite or biomarker associated with pesticide(s) and other agrochemicals with higher concentration	Concentration level compared to other international studies	Other identified pesticide and other agrochemicals contaminants metabolites	Pesticides, other agrochemical contaminants, and cancer according to IARC classification (1, 2A and 2B)	Carcinogenic, probably or possibly carcinogenic to humans (Group 1, 2A or 2B) allowed in Chile
Landeros et al., 2022 (20)	South-Central	Cross-sectional	Biomarker peripheral blood of rural agricultural workers exposed to pesticide mixtures (*n* = 30) and unexposed urban workers (*n* = 30)	Frequency of micronuclei (genetic damage)	High (genotoxicity compared to agricultural workers in Brazil, Ecuador, Bolivia and the Dominican Republic. In Europe, also high when compared to Greece)	N/A	Self-report to pesticides OP (diazinon and malathion 2A)	Diazinon and malathion
Mariottini et al., 2002 (106)	South-Central	Cross-sectional	Adult adipose tissue. Chile = 10 women; Siena, Italia = 7 women and 11 men	DDT	Low. compared to Italy	HCB and PCBs	DDT (2A), HCB (2B) and PCBs (1)	No
Muñoz-Quezada et al., 2012 (107)	South-Central	Longitudinal	Metabolites DAPs Urine children (*n* = 190) DAPs OP metabolites in urine	DEAPs and DMAPs (urine)	High. Compared to United States, Canada, Ecuador and Europa	N/A	Diazinon (2a)	Diazinon
Muñoz-Quezada et al., 2019 (108)	South-Central	Longitudinal	Children’s urine (*n* = 48) DAPs and OP-specific metabolites	DEAPs, DMAPs, TCPy, PNP	High. Compared to United States, Canada, Ecuador and Europa	Diazinon and malathion	Diazinon (2A), malathion (2A), and parathion (2B)	Diazinon, malathion
Muñoz-Quezada et al., 2020 (109)	South-Central	Longitudinal	Children urine (*n* = 48) OP specific metabolites, pyrethroids and herbicide 2,4-D	3-PBA and TCPy	High. Compared to United States, and Europa	Trans-DCCA, 2,4-D, and Diazinon	2,4-D (2B), diazinon (2A), parathion (2B), and malation (2A)	Diazinon, 2,4-D, and malathion
Ramírez-Santana et al., 2018 (110)	Central	Cross-sectional	AchE, BChE and APEH in human venous blood (EE = 66, OE = 87, NE = 100)	OP and carbamates	High (Exposure). Canada and United State	N/A	OP and carbamates (diazinon and malathion, 2A)	Diazinon, malathion
Zúñiga-Venegas et al., 2015 (111)	Central	Cross-sectional	PON1 in blood in humans (*n* = 85 EO; *n* = 60 EE; *n* = 33 NE)	Susceptibility to chlorpyrifos and other OPs	High. The levels of exposure to pesticides in the studied population can be considered high, especially due to the large interindividual variability and the observed genetic susceptibility.	N/A	OP (diazinon and malathion, 2A)	Diazinon and malathion
Zúñiga-Venegas et al., 2022 (112)	Central	Cross-sectional	AChE, BChE, PON1 in human venous blood (*n* = 85 EO; *n* = 60 EE; *n* = 33 NE)	Susceptibility to chlorpyrifos and other OPs	High (occupational and rural exposure)	N/A	OP and carbamates (diazinon and malathion, 2A)	Diazinon and malathion

DDT, dichloro-diphenyl-trichloroethane; HCB, Hexachlorobenzene; PCBs, Polychlorinated Biphenyls; OP, Organophosphates; DAPs, Dialkylphosphates; DEAPs, Diethyl alkylphosphates; DMAPs, Dimethyl alkylphosphates; TCPy, 3,5,6-Trichloro-2-pyridinol, this is a metabolite of chlorpyrifos, an organophosphate pesticide; PNP, Metabolite paranitrophenol is the commonly used abbreviation to refer to the specific metabolite Parathion methyl; IMPy, 2-isopropyl-6-methyl-4-pyrimidinol, a metabolite of diazinon; MDA, malathion on malondialdehyde a biomarker of oxidative stress and the antioxidant status in cancerous patients; Trans-DCCA, Trans-3-(2,2-dichlorovinyl)-2,2-dimethylcyclopropane carboxylic acid is part of the group of pyrethroids Pesticides; 2,4-D, 2,4-Dichlorophenoxyacetic acid, a synthetic herbicide used to control broadleaf weeds in agriculture; AchE, Acetylcholinesterase activity is a well-known biomarker for exposure to organophosphate or carbamate compounds; BChE, biomarker associated with the enzyme butyrylcholinesterase, also known as pseudocholinesterase; APEH, Acyl Peptide Enzyme Hydrolase, serves as a sensitive biomarker for detecting acute low-level exposure to organophosphates (OPs); PON1, Paraoxonase 1, an enzyme that can hydrolyze certain organophosphates and has a protective role against their toxicity; EE, Environmentally exposed; EO, Occupationally exposed; NE, Not exposed; N/A, Not Applicable.

* PCBs, PAHs, HCB, and PBDEs are not pesticides, but persistent organic pollutants that enter the environment through similar pathways. Their inclusion in the table aims to provide a more comprehensive view of toxic chemical exposure in agricultural systems, as they can interact with pesticides and increase risks to human health and the environment.

The evaluation of high or low pesticide levels was conducted by comparing the concentrations reported by each study with these established standards. Additionally, the assessment took into account the levels identified as high or low by the authors of each manuscript, based on their review of previous studies. Concentrations above the allowable limits or those identified as high by the study authors were classified as high, indicating potential health risks, while those below the limits or identified as low were classified as low.

## Results

After analyzing the 12 searches across the three databases, an initial set of 7,318 articles were identified. After eliminating duplicates, this number decreased to 2,790 articles. Subsequently, the selection process narrowed down the results to a final set of 86 manuscripts through the rigorous application of inclusion and exclusion criteria. Of these, 83 complete manuscripts were included in the qualitative synthesis (Figure 1).

**Figure 2:**
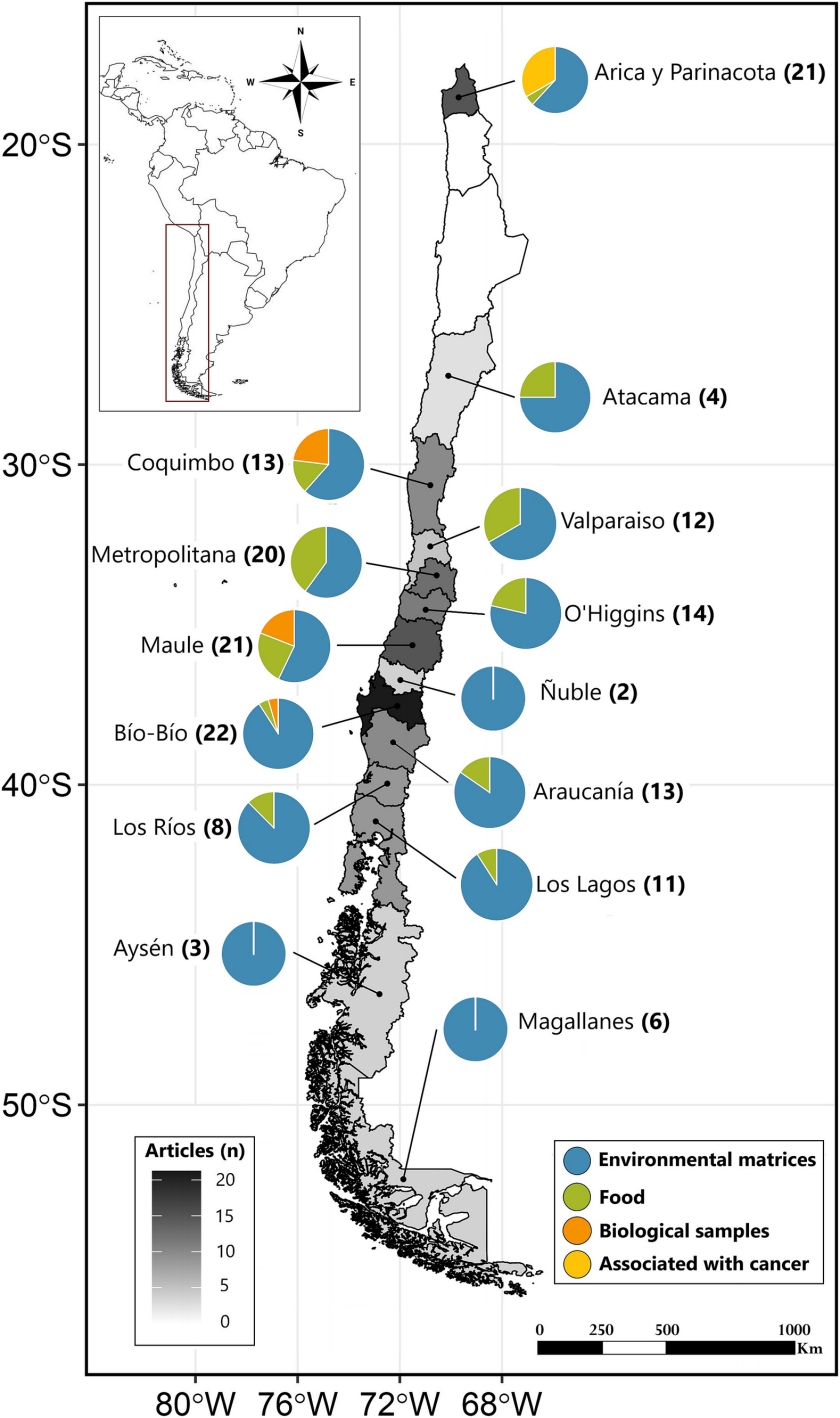
Geographical distribution of the studies covering pesticide exposure in Chile (1996–2024) by regions, environmental matrices, foods, human biological samples, and cancer associations (self-developed)*. * Geographic distribution of epidemiological research assessing pesticide exposure in Chile (1996–2024). The grayscale map reflects the frequency of regional mention based on the sample location (also in parentheses). The accompanying pie charts delineate the distribution of studies across research categories: “Environmental matrices” (blue), “Food” (green), “Human biological samples” (orange), and “Associated with cancer” (yellow) (see methods for details). Some papers refer to more than one region; hence the sum of the regional values does not correspond to the total number of papers included in the analysis (84). Three of the 83 studies were not included in this map as they do not report a more specific geographic reference to locate their occurrence (40, 42, 43, 61).

The 83 selected studies covered a period from 1996 to 2024, with 43% published in the last ten years. Detailed geographical distribution of these studies within the country is presented in Figure 2.

In Chile, macrozones are defined as geographic divisions that help organize and analyze regions with similar geographical characteristics, aiding the management and analysis of regional data and policies. These divisions typically include the country’s Northern, Central, Metropolitan, South-Central, South, and Southern areas (36).

Some studies evaluated more than one macrozone or region, so the percentage describing where the studies were conducted does not add up to 100%. This is because certain studies covered multiple regions to provide a broader view of exposure and its effects.

Most studies (36%) were exclusively conducted in the South-Central macrozone, which encompasses the regions of O’Higgins, Maule, Ñuble, and Bío Bío. Approximately 12% of the studies focused exclusively on the Northern macrozone, comprising the Arica and Parinacota, Tarapacá, Antofagasta, and Atacama regions. In the Central zone (Coquimbo and Valparaíso), studies accounted for 11%, while a similar proportion was dedicated solely to the Metropolitan area. In the South macrozone (La Araucanía, Los Lagos, and Los Ríos), 12% of the studies were conducted. Meanwhile, 8% of the research was confined to the Southern zone (Aysen, Magallanes, and the Chilean Antarctic territory). Lastly, 20% of the studies spanned multiple macrozones, while a minimal 1% did not specify their geographic area of focus.

Among the pesticide(s) and other agrochemicals contaminants evaluated, 20% of the reviewed studies reported a significant occurrence of chlorinated compounds, including organochlorines (OCs) and polychlorinated biphenyls (PCBs). Although PCBs are not classified as pesticides, their recurrent detection as xenobiotic contaminants in pesticide formulations warrants their consideration. The historical application of PCBs in agro-industrial practices has resulted in their environmental recalcitrance and potential chemical interactions with other agrochemicals, amplifying ecotoxicological and public health risks (37, 38).

Furthermore, 71% of the studies exhibited high concentrations pesticide(s) and other agrochemicals contaminants that may imply a health risk, with recommendations based on established standards or in comparison to residue levels found in other international research. It is necessary to highlight that 79.5% of the studies identified one or more chemical agent classified as carcinogenic according to the IARC classification (group 1 = 30 studies; group 2A = 46 studies; group 2B = 40 studies).

### Studies on pesticide and other agrochemicals contaminants exposure in environmental matrices

Supplementary Table 1 provides a comprehensive summary of studies investigating the presence and concentration of pesticides and other agrochemicals contaminants across diverse environmental matrices in various regions of Chile. Each entry includes details such as the author and publication year, macrozone (region), study design (longitudinal or cross-sectional), specific pesticides found, concentrations reported, and additional identified chemicals in the same matrix sample. The IARC classification was employed to categorize pesticides and other agrochemicals permitted in Chile as probably or possibly carcinogenic to humans.

Studies examining exposure to pesticides and other agrochemical contaminants in environmental matrices represented 69% of the total research reviewed (57 out of 83) and are summarized in Supplementary Table 1. Of these, 51% (29 out of 57) were conducted in the South-Central macrozone of Chile. Regarding the study designs for environmental research, 79% adopted a longitudinal design, 4% focused on measurement technique validation, and 17% had a cross-sectional design. Among the environmental matrices, the most frequently studied were freshwater systems (23%), animal by-products and waste (32%), sediment and soil (17%), air (17%), while 11% of the studies evaluated samples of sea water or marine sediment.

The most frequently detected pesticides and agrochemical contaminants were PCBs (*n* = 23), hexachlorocyclohexanes (HCHs, *n* = 17), dichlorodiphenyltrichloroethane (DDT, *n* = 23), and chlorpyrifos (*n* = 9). Among the 57 studies, 45 (79%) reported the presence of one or more pesticides or agrochemical contaminants classified as probably or possibly carcinogenic to humans according to IARC (group 1 = 29 studies; group 2A = 30 studies; group 2B = 29 studies). Additionally, eight studies identified specific pesticides within these categories, including diazinon, 2,4-D, malathion, and diethanolamine, which are still authorized for use in Chile.

A significant proportion (67%) of the detected pesticide and other agrochemical contaminants levels in these studies were considered to have high concentrations of residues. These assessments were made by the authors in the discussion of the manuscripts, comparing the levels with national standards or international studies (39–96).

For example, Baldassin et al. (39) studied *Magellanic penguin* liver samples from the Southern region, identifying low concentrations of DDT alongside other agrochemical contaminants such as HCBs, Drins PCBs, and PBDEs. Similarly, Balsebre et al. (40) collected *honey bee* samples from the South-Central region, uncovering elevated chlorpyrifos levels along with other insecticides such as fipronil, thiamethoxam, acetamiprid, acrinathrin, metamidophos, dimethoate, diazinon, methidathion, profenophos, azinphos-methyl, and coumaphos. These studies shed light on the pervasive presence of harmful chemicals in the environment, providing valuable insights for policymakers to enact regulations safeguarding human health and wildlife populations.

### Pesticide exposure studies in ready-to-eat foods

The following compilation (Table 2) presents studies conducted on various types of food in Chile (76, 96–105), examining the presence and concentration of pesticides. Each study encompasses essential details such as the author, publication year, macrozone (region), study design (longitudinal or cross-sectional), type of food analyzed, pesticides identified with concentrations, other detected chemicals or pesticides, and whether these pesticides fall under IARC classifications as possibly carcinogenic (categories 1, 2A, or 2B), along with their status of allowance for use in Chile.

Thirteen percent (*n* = 11) of the total research was focused on measuring pesticide residues in food matrices intended for human consumption, such as vegetables, fruits, and oil (see Table 2). These food-related studies were primarily conducted in the Metropolitan region, representing 73% of the total (*n* = 11). Out of these, 54.5% adopted a longitudinal design, 18% had an experimental focus, and 27% were based on a cross-sectional design. Notably, 64% of the food matrices evaluated were vegetables intended for human consumption.

When analyzing pesticide residues in food, the compounds with the highest reported concentrations were chlorpyrifos and metamidophos. Chlorpyrifos was present in five out of the eleven studies.

Among the various reports, we highlight a study by Concha-Meyer et al. (96) investigating frozen fruits and vegetables from the South-Central region, revealing elevated levels of iprodione and two forms of spinosad pesticide. Another study by Elgueta et al. (97) focused on lettuce, chard, and spinach from the Central and Metropolitan regions, detecting high levels of methamidophos pesticide, and on the other hand, several others categorized by IARC as possibly carcinogenic. These studies highlight potential health risks associated with consuming foods containing harmful chemicals, particularly certain pesticides. It is essential to note that while not all studies provide evidence of harm from exposure to these substances, they raise concerns about the cumulative effects over time when regularly consumed through diets.

It is important to highlight that in 64% of the studies, high concentrations of pesticide residues were identified in the evaluated foods, potentially posing health risks. These findings were reported by the study authors (96–105).

Regarding pesticide residues classified as probably or possibly carcinogenic according to the IARC classification, they were found in six of the studies (group 2A = 4 studies; group 2B = 3 studies). Most of these compounds fell into Group 2A, and it is relevant to note that they are not prohibited in Chile.

### Studies with pesticides measured in biological samples of the Chilean population

This section presents various studies exploring the effects of pesticides on human samples, each providing comprehensive details such as the author, publication year, macrozone, study design, population, sample size, biomarker or metabolite associated with pesticide exposure, concentration levels compared to international studies, other identified pesticides or chemical metabolites in the samples, and their classification as probably or possibly carcinogenic to humans by IARC (see Table 3).

For instance, Landeros et al. (20) investigated the genetic damage caused by pesticide exposure in rural agricultural workers (*n* = 30) compared to unexposed urban workers (*n* = 30). They detected a high frequency of micronuclei, indicating genotoxicity, and established a relationship between genetic damage and reproductive problems in the exposed workers (20). Mariottini et al. (106) analyzed adipose tissue for DDT residues in adults from Italy and Chile, revealing higher DDT concentrations in Chile. Muñoz-Quezada et al. (107–109) conducted longitudinal studies examining children’s urine samples for organophosphates (OP) specific metabolites, suggesting significant exposure to dangerous pesticides.

Ramírez-Santana et al. (110) investigated blood enzyme activity related to OP and carbamate herbicide exposure, detecting significant exposure among occupational groups. Zúñiga-Venegas et al. (111, 112) conducted cross-sectional studies assessing blood enzyme activity affecting susceptibility to chlorpyrifos and other pesticides.

When examining research focused on assessing exposure, susceptibility, and effect biomarkers in biological samples of the Chilean population, mostly within the scope of observational studies, we identified that they accounted for 11% (8 out of 83) of the total studies analyzed, as summarized in Table 3.

Most of these investigations were conducted in the Central-South zone of Chile, representing 62.5% of the cases. Five out of the eight studies adopted a cross-sectional study design, while the rest were longitudinal in nature.

On the other hand, six studies focused on evaluating biomarkers of pesticide exposure in adult human blood, including AChE and BchE (110–112), OP susceptibility through the Q198R and L55M Paraoxonase (PON1) polymorphisms (111, 112), and genotoxicity due to OP exposure (20). Additionally, research was conducted to assess the presence of OCs and PCBs in human adipose tissue, revealing lower concentrations compared to a sample from Italy (106).

Regarding studies on dialkylphosphorated and specific OP metabolites, pyrethroids, and 2,4-D in urine, as well as blood biomarkers activity like AChE, BChE, and PON1 polymorphisms; 6 out of 8 studies identified elevated concentrations that could have health implications compared to international studies reported by the authors of the reviewed manuscripts (20, 107–110, 112). Following the IARC classification, one study found at least one metabolite related to group 1 pesticides, eight studies found metabolites linked to group 2A pesticides, and three studies within group 2B. Additionally, 75% of these studies (*n* = 7) detected the presence of some pesticide metabolites considered probably or possibly carcinogenic to humans, such as diazinon, malathion, and 2,4-D, which are not prohibited in Chile.

### *In vitro* studies (cell cultures exposed to pesticides) and human carcinogenicity

This compilation (Table 4) presents a series of studies delving into the impact of various pesticides on human breast cells. Each study encompasses details on the author, publication year, location (all were conducted in the Northern macrozone), experiment type (experimental/human breast cells *in vitro*), intervention (incubation with varying pesticide concentrations), and outcome evaluation (cellular lethality or transformation), scrutinizing changes in cell viability, proliferation, gene expression, growth capacity, and invasive characteristics.

**Table 4:** Summary of studies with exposure, effect, and susceptibility biomarkers and other agrochemicals* in biological samples of the Chilean population (*n* = 8).

Author and year of publication	Macrozone	Design	Intervention	Outcome evaluation	Outcome	Exposure concentration*	Pesticides and cancer according to IARC classification (1, 2A and 2B)	Carcinogenic, probably or possibly carcinogenic to humans (Group 1, 2A or 2B) allowed in Chile
Cabello et al., 2003 (113)	Northern	Experimental/Human breast cells *in vitro*	Incubated MCF cells with varying concentrations of malathion in DMEM containing 10% of fetal bovine serum	Cellular lethality for each experimental condition, for the lethal dose (LD) as a percentage of cells totals.	The cells incubated with high doses of malathion, produce alterations in the Cell viability of human breast carcinoma.	64 μg/mL and 128 μg/mL	Malathion (2A)	Malathion
Calaf et al., 2007 (114)	Northern	Experimental/Human mammary epithelial cells *in vitro*	MCF-10F cells growing in lethal diploid human mammary epithelial cell lines with parathion.	MCF-10F cell line proliferation for each treatment group	Parathion affects epithelial cells in the human breast and is an initiating factor in the transformation process of breast cancer.	100 ng/mL	Parathion (2B)	No
Calaf et al., 2007 (115)	Northern	Experimental/Human mammary epithelial cells.	MCF-10F cells growing in human breast epithelial cell lines with the OP parathion	Alteration of human genes for substance metabolism	Parathion in the presence of estradiol induces changes in the expression of the human metabolism gene in mammary cells.	100 ng/mL + 10^−8^ M estradiol	Parathion (2B)	No
Calaf et al., 2008 (116)	Northern	Experimental/Mammary epithelial cells humans i*n vitro*	Spontaneously immortalized MCF-10F cells that grow in line epithelial cells of the human chest with parathion and malathion	Transformation malignant evidenced by the ability of independent growth from anchorage and invasive features	Estrogen with parathion alter the expression of proteins in the transformation of breast cells *human in vitro*	100 ng/mL + 10^−8^ M estradiol	Parathion (2B) and malathion (2A)	Malathion
Calaf et al., 2008 (117)	Northern	Experimental/Human mammary epithelial cells *in vitro*	MCF-10F cells grown in human breast epithelial cell lines with the OP parathion and E2, separately or in combination	Malignant transformation evidenced by anchorage-independent growth capacity and invasive characteristics. Analysis of the genes expression.	OP parathion in high doses has the potential to cause malignant transformations in breast cells	100 ng/mL + 10^−8^ M estradiol	Parathion (2B)	No
Calaf et al., 2009 (118)	Northern	Experimental/ Mammary epithelial cells humans *in vitro*	Spontaneously immortalized MCF-10F cells that grow in line epithelial cells of the human chest with parathion and malathion.	Transformation malignant evidenced by the ability of independent growth from anchorage and invasive characteristics.	Estrogen, parathion and malathion cause malignant transformation and genomic instability in mammary cells.	Combination of 100 ng/mL of parathion, malathion, and estradiol	Parathion (2B) y malathion (2A)	Malathion
Muñoz et al., 2023 (119)	Northern	Experimental/Human mammary epithelial cells	Estrogen-sensitive breast cancer cell lines treated with glyphosate	Glyphosate mimicking the cellular effects of 17β-estradiol	Glyphosate at high concentrations induces estrogen-like effects through an ERα ligand binding site-dependent mechanism.	1×10^−2^ M	Glyphosate (2A)	Glyphosate

DMEM, modified eagle medium; MCF-10F, spontaneously immortalized nontransformed human breast epithelial cell line that does not grow in soft agar or form tumors in nude mice.

* The pesticide concentrations used in the experimental studies, as shown in this table, are generally higher than those typically found in the environment. These concentrations are selected to assess maximum toxic effects and do not necessarily reflect the doses to which populations are chronically exposed in agricultural settings. Additional studies are needed to extrapolate these results to lower and prolonged environmental exposure.

These investigations specifically focus on pesticides such as malathion and parathion, both classified by IARC as probably or possibly carcinogenic to humans (113–118). Glyphosate, another pesticide studied, exhibits cellular effects mirroring those of 17β-estradiol (119). The collective findings indicate that these pesticides, particularly when combined with estrogen, induce genomic instability and malignant transformations in mammary cells. In summary, these studies illuminate the potential deleterious effects of certain pesticides on human breast cells.

Of the total set of studies, 9.5% (*n* = 7) corresponded to experimental research carried out in laboratories using human cells. It is worth mentioning that these studies were conducted in the Northern macrozone of the country, specifically in the Arica and Parinacota Region. Moreover, all experiments used human breast cells as the study material.

Regarding the substance under investigation, 1 out of 7 studies administered different doses of malathion in the cell line (113), three studies evaluated the effect of parathion in cells (114–117), and two publications applied doses of both malathion and parathion (116, 118). Only one study used doses of glyphosate in their experiments (119).

The results of these Chilean *in vitro* studies suggest that high levels of exposure to parathion, malathion, and glyphosate could be associated with the development of breast cancer, as presented in Table 4.

## Discussion

The results of the analyzed scientific evidence reveal concerning findings regarding pesticide or other agrochemicals contaminants exposure in Chile, primarily due to the presence of contaminants that have been banned in the country, such as persistent organic compounds, including organochlorine pesticides.

Although this article primarily focuses on pesticide exposure, it is important to highlight those other environmental contaminants (Supplementary Table 1), such as polychlorinated biphenyls (PCBs), polycyclic aromatic hydrocarbons (PAHs), hexachlorobenzene (HCB), and polybrominated diphenyl ethers (PBDEs), are also present in agricultural and rural systems. These compounds, while not pesticides, can enter agricultural environments through soil, water, and air contamination, and some may be found in products used in agriculture. Due to their persistence in the environment, PCBs and HCB, among others, can accumulate and lead to prolonged exposures, particularly in rural areas. Their coexistence with pesticides in these environments increases the risk of adverse health effects, such as the potentiation of combined toxicities. This underscores the need for a comprehensive risk assessment that considers both pesticides and these persistent environmental contaminants (17).

Numerous reported cases indicate that the SAG intercepts the entry of prohibited pesticides in the border regions with Bolivia, Peru, and Argentina or Chilean ports (120, 121). These illegal activities are the most probable contributors to the entry of dangerous products already prohibited in Chile. This could explain the observed residues of these banned substances in humans, vegetables, and various environmental matrices. When these compounds combine with other contextual risk factors, they could significantly impact the development of certain types of cancer (8, 122, 123). From a population health perspective, where environmental determinants of health are essential to understanding the causes of diseases, environmental quality, as measured through air, soil, water, and food quality, is essential in guiding population-wide preventive health actions. Thus, a significant proportion of Chilean environmental studies demonstrate the presence of pesticides that can come into contact with people through air, soil, and water. These findings are relevant nationwide and span a vast research period (1996 to 2023). The analyses reported here indicate environmental contamination, posing an increased exposure risk for the population. One of the key concerns is that many pesticides detected in these studies are classified as probably or possibly carcinogenic to humans by the IARC (16). In Chile, chemicals such as diazinon, difenilamina, chlorothalonil, malathion, and diethanolamine, which are restricted in the European Union and the USA for agricultural use, remain authorized (21, 22, 31). However, progress has been made in Chile with the recent prohibition of glyphosate-based products containing polyethoxylated tallowamine (21), a step beyond EU regulations (25), which recently extended glyphosate’s approval until December 15, 2033, based on assessments considering no significant risks to human health despite its classification by the IARC as probably carcinogenic to humans. This aligns with critiques of regulatory processes that often assess glyphosate in isolation, ignoring the toxicity of co-formulants such as polyethoxylated tallowamine, which have been shown to significantly amplify its harmful effects (124). These regulatory discrepancies highlight the need to continuously update national policies in line with emerging evidence to protect public health. Furthermore, international research, including agricultural cohort and case–control studies, has consistently linked exposure to pesticides such as pyrethroids, herbicides, or organophosphates to higher risks of various cancers, including acute lymphocytic leukemia, childhood brain tumors, breast cancer, and prostate cancer (125–131).

The review identified several pesticides linked to cancer in humans, many of which have been classified as carcinogenic to humans by the IARC. For example, malathion, diazinon, and glyphosate are classified as “probably carcinogenic to humans” (Group 2A) due to limited evidence in humans and sufficient evidence in animals. Additionally, parathion is classified as “possibly carcinogenic to humans” (Group 2B) based on similar criteria. These classifications are supported by studies showing associations with cancers such as non-Hodgkin’s lymphoma, leukemia, and mammary gland tumors. Mechanistic studies also reveal DNA damage, oxidative stress, and hormonal disruption as contributing factors to these outcomes (132).

However, much of the evidence comes from studies that used high-dose exposures, which may not accurately represent the chronic, low-dose exposures typical of agricultural environments. To better understand the health risks of long-term, low-dose exposure to pesticides, particularly in rural populations exposed to residues over time, further research is essential, focusing on realistic environmental exposure levels (133, 134). Additionally, there is a need to explore mitigation strategies, including stricter regulations, enhanced safety protocols for farmworkers, and the development of safer pesticide alternatives.

According to the reviewed studies, local or regional sources of contamination by OC pesticides are identified, mainly associated with industrial or agricultural areas (South-Central macrozone). These sources are linked to the persistence of these pesticides in soil and sediment in the Central Valley basins, as well as to dispersion in nearby areas in the so-called environmental drift caused by waste burning or high temperatures during warm months, in which these toxic substances can drift over areas close to schools or other areas where people live or work (66, 72).

Exposure to pesticides through food intended for human consumption is another highly complex issue. Pesticide residues in vegetables and fruits are a matter of great concern since these foods constitute a fundamental part of the population’s diet. The detection of many chemical compounds and, in some cases, levels exceeding the Maximum Limits of Pesticide Residues (MLPRs) established by the Chilean Ministry of Health (35) based on the Maximum Residue Limits (MRLs) from of *Codex Alimentarius* (32) for fruits and vegetables poses significant health risks for consumers (99, 100). This risk becomes even more critical when it comes from pesticides classified as potential carcinogenic to humans, as they can induce adverse effects on individuals susceptible to the disease (135), despite the limited available evidence (136).

Studies examining the consumption of vegetables containing pesticide residues suggest that chronic health risks are generally low compared to the significant benefits of consuming fruits and vegetables, showing that these benefits often outweigh the potential risks associated with cancer (137–139). However, it is crucial not to underestimate risk estimates, particularly considering individual susceptibility. Persistent uncertainties, notably concerning children, highlight higher health risks associated with the consumption of fruits and vegetables containing pesticide residues such as chlorpyrifos, diazinon, dimethoate, and metamidophos, among others (76, 135, 139).

Therefore, it is desirable to reduce exposure by prohibiting these pesticides in fruits and vegetables while promoting a rich and varied diet with these foods. Controversially, many of the pesticides detected in Chilean food items are highly regulated in the destination countries of the main plant-origin products exported to those markets (140). Therefore, producing fruits and vegetables for exports requires high care on MRLs and prohibited substances, which might not be accurate for internal markets for several reasons.

Exposure from various sources and routes, as observed in agricultural populations, becomes a relevant factor when evaluating the multifactorial components of pesticide exposure and other chemicals concerning the development of chronic diseases such as cancer in the population (141, 142).

Despite Chile having established regulations in line with international standards (25, 124), such as MLPRs in food according to technical regulation following the MRLs from the *Codex Alimentarius* (32, 35), the compiled data reaffirm the urgent need to strengthen current monitoring mechanisms, along with other essential public health actions such as inspections and the development of more ambitious food surveillance programs.

This finding is of outstanding relevance in safeguarding the population’s health from exposure to these chemical agents and ensuring proper compliance with existing regulations. In this context, environmental and biological monitoring in at-risk populations, both in the case of children and exposed workers, becomes an essential tool for the timely detection of irresponsible or illegal practices in the sale and use of pesticides.

In addition to environmental and food studies, research involving biological biomarkers of exposure conducted on the Chilean population confirms worrisome contamination levels within individuals. The detection of pesticide metabolites in children’s urine (107–109) and the presence of effect biomarkers in adults’ blood (110–112) indicate that pesticide exposure affects both the occupationally exposed population and people residing in rural areas nearby farming activities. The exposure levels identified pose significant risks to the affected groups (109).

Research on pesticide exposure in human populations, especially regarding cognitive impairments and genotoxicity, has been limited (112). Studies in the Coquimbo and Maule regions have highlighted a significantly increased risk of genotoxicity in agricultural workers exposed to pesticides, particularly OP (20, 111, 112).

These findings support the association between pesticide exposure and genetic damage, suggesting an increase in oxidative stress that impairs the body’s ability to metabolize organophosphate (OP) pesticides. This ability diminishes with prolonged exposure, thereby increasing the risk of chronic diseases such as cancer (112). Additionally, the epigenetic dimension has raised growing concerns, as pesticides can induce epigenetic changes in DNA, affecting critical processes related to carcinogenesis (15, 143, 144). Pesticide exposure, including banned compounds, raises concerns about long-term epigenetic effects and the possibility of transmitting inherited epigenetic modifications to future generations, thereby increasing the risk of cancer (145).

While research on this topic in Chile remains limited, the significance of the already-acquired results is undeniable. Investigating the underlying epigenetic mechanisms and their role in cancer development is imperative. A sustained exploration of this theme will aid in developing evidence-based prevention strategies, ultimately mitigating risks for the Chilean population.

Specifically, national research on the association between cancer and pesticide exposure has been based on *in vitro* experimental studies in cell lines, providing additional information about the potential relationship between exposure to malathion and glyphosate and the process of carcinogenesis in breast tissue (113–119). However, more advanced studies are required to determine the necessary dose–response, establish early damage markers, and identify the involved pathophysiological mechanisms, considering the country’s diverse production and geographical areas.

In agricultural settings, pesticide exposure concentrations can vary significantly depending on the type of pesticide, the mode of application, and environmental factors such as wind, temperature, and soil type. Typically, environmental concentrations range from nanograms to micrograms per liter (ng/L to μg/L) in water and micrograms per cubic meter (μg/m^3^) in the air. For example, airborne pesticide concentrations in agricultural environments can reach up to 10–100 μg/m^3^ immediately after pesticide application, but they decrease over time as the chemicals disperse and degrade (146, 147).

When comparing these environmental exposure levels to the concentrations used in *in vitro* studies (113), such as the 64 μg/mL and 128 μg/mL reported by Cabello et al. (113), it’s evident that laboratory experiments often use higher doses than what would typically be encountered in real-world agricultural exposures (33, 148). These higher concentrations are designed to assess potential toxic effects under worst-case scenarios, but translating these results to low-level, chronic environmental exposures requires careful consideration.

The collected information has significant implications for public health and highlights the need to review and update pesticide regulations in Chile (149). The presence of pesticides in food and the environment, especially those classified as possibly carcinoginc to humans, demands stricter measures to protect health, such as banning and restricting the use of chemical agents associated with certain types of cancer. It is imperative to minimize environmental exposures, especially during children’s growth and development stages, as supported by scientific evidence.

When reviewing the concentrations of OP pesticide metabolites in urine from Chilean studies (107, 108), such as dialkylphosphates (DAPs) (including OP metabolites); chlorpyrifos and diazinon (insecticides that also generate DAPs metabolites diethylphosphate and diethyldithiophosphate); and methyl parathion (an insecticide that also produces DAPs metabolites dimethylphosphate, dimethyldithiophosphate, and dimethyldihiophosphate), as well as non-specific pyrethroid metabolites (3-PBA and trans-DDCA) (109), and the activities of blood enzyme biomarkers AChE, BChE and PON1 (110–112); and by comparing these results with other international studies that evaluated the association between cancer development and exposure to these pesticides using the same biomarkers in populations of similar origin, it was observed that in the Maule (20, 107–109) and Coquimbo (110) regions, the medians, means, or frequencies were similar to other cases reported in international studies showing an association with some types of cancer (126, 129, 150, 151).

Not only is exposure to OP insecticides such as methamidophos or chlorpyrifos a concern, with recent efforts in Chile to prohibit and restrict their use (152, 153), but also the evidence from international studies confirming the association between childhood cancer and early exposure to pyrethroid pesticides (126, 129), which are still sold in Chile without restrictions or public education programs.

In Chilean studies, children residing in rural areas, face multiple exposures to these chemical agents (19, 107–109, 112). This exposure is due to their proximity to agricultural fields where pesticides are used to control pests in vegetable and fruit crops, as well to consuming fruits contaminated with multiple residues. Additionally, indoor exposure occurs, for example, through insect control during the summer, gardening activities, and the use of pesticides for mite control in pets and for treating pediculosis in children. In other words, children experience a variety of sources and pathways of pesticide exposure. This complex situation, combined with the possibility of genetic susceptibility of the Chilean population to OP pesticides (13), amplifies the risk factors that may contribute to the long-term development of non-communicable chronic diseases, such as cancer, and other potential adverse health consequences (141).

The review conducted exhibits certain limitations that warrant attention. The information search was limited to three principal databases, potentially omitting valuable studies from lesser-known or regional sources not indexed in Scopus, Web of Science, or PubMed. This limitation could introduce bias by favoring findings from more established sources and neglecting research from regional publications or specialized databases. Furthermore, there was no search for grey literature or unpublished reports, which are significant data sources, particularly in public health and policy-making domains. They are vitally important in developing countries, where some pertinent studies may not achieve international visibility.

Although the review provides a comprehensive and varied overview of existing research, there was no assessment of the methodological quality of the included studies.

Nevertheless, this approach was selected because the research objective was to examine a broad and complex area, such as the relationship between pesticide exposure and its potential carcinogenicity (28). This approach facilitates the identification of main trends and research gaps, and the formulation of broad recommendations for future studies and policy development.

Finally, it is mandatory to promote further multidisciplinary research on the relationship between pesticide exposure and cancer in the Chilean population. This recommendation includes conducting cohort and case–control epidemiological studies to better understand the risks and implement more effective preventive measures, as has begun in the Maule region (154). The evidence reviewed and analyzed provides a comprehensive view of Chile’s pesticide exposure and various exposure scenarios. Although associations with cancer have not been established at a general or specific level, their presence in the environment poses a threat to the health of at-risk populations in Chile. The findings highlight the importance of effectively addressing this public health issue and promoting safer and more sustainable agricultural practices that can be applied throughout Latin America (13).

Stricter regulation, public awareness, and environmental and occupational education programs directed at farmers and rural communities are critical to protecting the region’s health and environment.

## Conclusion

The review documents the presence of pesticides in Chile across environmental matrices, food, and human populations, including several compounds classified as probably or possibly carcinogenic to humans by the IARC. While the evidence does not establish definitive links between pesticide exposure and cancer, it highlights exposure levels that often exceed safety thresholds, particularly in agricultural settings and among occupational groups.

Key findings point to genotoxic effects and the potential for epigenetic alterations, which warrant further investigation. Regulatory gaps, particularly concerning pesticides classified as carcinogenic, underline the need for updated policies and improved enforcement mechanisms.

Future research should prioritize long-term epidemiological studies and the evaluation of epigenetic impacts of pesticide exposure. Strengthened monitoring systems and educational programs targeting rural communities and agricultural workers are also necessary to reduce exposure and associated health risks. These actions can help address current gaps and inform safer agricultural practices.
