# Supplementary material for: Exposure to pesticides in Chile and its relationship with carcinogenic potential: a review
Source: Front Public Health. 2025 Apr 1;13:1531751. doi: 10.3389/fpubh.2025.1531751 (PMC11996790; doi:10.3389/fpubh.2025.1531751)
Supplement: Supplementary file 1 [file Supplementary_file_1.docx]

**Supplementary Table 1.** Summary of studies related to pesticide exposure and other agrochemicals* in environmental matrices in the Chilean territory (n = 57)

| **Author and year of publication** | **Macrozone** | **Design** | **Environmental matrix** | **Pesticide(s) and other agrochemicals contaminants with higher concentration** | **Concentration level as reported by each study reviewed (compared with international or national standards)** | **Other identified pesticides and other agrochemicals contaminants (e.g., PCBs, PBDEs, PAHs, HCB, HA, and others)** | **Pesticides and other agrochemicals contaminants according to IARC classification (Group = 1, 2A and 2B)** | **Carcinogenic, probably or possibly carcinogenic to humans (Group 1, 2A or 2B) allowed in Chile** | |
| --- | --- | --- | --- | --- | --- | --- | --- | --- | --- |
| Baldassin et al., 2016 (38) | Southern | Longitudinal | Magellanic penguin liver (animal by-products and waste) | DDT | High. Compared to levels detected in Brazil, Uruguay, and Antarctica (PCBs) | HCB, Drins, PCBs and PBDE | DDT (2A), HCB (2B), aldrin/dieldrin (2A), and PCBs (1) | No | |
| Balsebre et al., 2018 (39) | South-Central | Cross-sectional | Honey bees (animal by-products and waste) | Chlorpyrifos | High. Compared to levels detected in Uruguay, France, Egypt, Poland, Greece, and France | Fipronil, thiamethoxam, acetamiprid, acrinathrin, methamidophos, dimethoate, diazinon, chlorpyrifos, methidathion, profenophos, azinphos-methyl, and coumaphos | Diazinon (2A) | Diazinon | |
| Barra et al., 2001 (40) | South-Central | Longitudinal | Land and lake water (freshwater systems) | DDT | High. Historical comparison in the same country from 1942 to 1996 and compared with lakes in Canada | 15 PAHs, 7 PCBs, 3 and OCs | DDT (2A) and PCBs (1) | No | |
| Barra et al., 2004a (41) | South-Central | Longitudinal | Marine sediment | Hexachlorocyclohexane | Low. Historical comparison in the same country from 1960 to 2001 and not detectable. greater concentration in areas of the Baltic Sea. | PCBs, HCHs, 137Cs, and organic carbon | PCBs (1) and HCHs (2B) | No | |
| Barra et al., 2004b (42) | Northern | Cross-sectional | Soil and lake sediment | PCBs | High  Historical comparison in the same country from 1960 to 2001 and ake sediments from the temperate northern hemisphere. | Pb, and organic carbon | PCBs (1) | No | |
| Barra et al., 2005 (43) | South-Central | Cross-sectional | Soil, river water, and snow (freshwater systems) | HCHs | High. Compared to levels detected in Lake Baikal and in the  Upper Great Lakes | PCBs, DDE, DDT, and PAHs | HCHs (2B), PCBs (1) and DDT (2A) | No | |
| Borghini et al., 2005 (44) | Northern, South-Central, and Southern | Longitudinal | Soil and lake sediment | HCB | Low. Compared with Germany, Poland and Austria | DDE, DDT, PCBs, and HCHs | HCB (2B), DDT (2A), PCBs (1) and HCHs (2B) | No | |
| Bridi et al., 2018 (45) | South-Central | Longitudinal | Raw honey (animal by-products and waste) | Acetamiprid | Low. Compared with the maximum residue limits for neonicotinoids in honey in the European Union | Imidacloprid, thiamethoxam, and thiacloprid | No | No | |
| Cárdenas-Soracá et al., 2020 (46) | South-Central | Validation | River water (freshwater systems) | 2,4-D | High. Compared to levels detected in Australia and United States | Atorvastatin, carbamazepine, cotinine, diuron, hexazinone, nicotine, hydroxycotinine, paracetamol, paraxanthine, and simazine | 2,4-D (2B) | 2,4-D | |
| Cárdenas-Soracá et al., 2019 (47) | South-Central | Validation | Sea water | HCHs | High. Compared with HCHs levels in Europe and North America | Lindane, HCHs (hexachlorocyclohexane), aldrin, dieldrin, DDE, endrin, DDD, DDT, and methoxychlor | HCHs (2B), lindane (1), aldrin/dieldrin (2A), and DDT (2A). | No |  |
| Climent et al., 2018 (48) | South-Central | Longitudinal | River water (freshwater systems) | Simazine and triazines | High. Compared with water levels in the European Commission and the United States. | Terbuthylazine, simazine, atrazine, DIA, DEA, DET, pyrimethanil, cyprodinil, kresoxim-methyl, metalaxyl, diazinon, diazoxon, and chlorpyrifos | Diazinon (2A) | Diazinon | |
| Climent et al., 2019a (49) | South-Central | Longitudinal | Air | Ethyl chlorpyrifos | High. Compared with Italy, Czech Republic, United States, and Canada | Lindane, HCHs, diazinon, pyrimethanil, penconazole, and malathion | Lindane (1), HCHs (2B), diazinon (2A), and malathion (2A) | Diazinon and malathion | |
| Climent et al., 2019b (50) | South-Central | Longitudinal | River water (freshwater systems) | Acephate (dissolved phase in 2015), diuron and pyrimethanil (dissolved phase in 2016), and triazine (particulate matter in both years) | High. Compared with regulations in the United States and Europe, and with Brazil | Chlorpyrifos oxon, chlorpyrifos, diazinon, pirimicarb, methidathion, imidacloprid, metamidophos, metalaxyl, azoxystrobin, tebuconazole, cyprodinil, myclobutanil, fludioxonil, HA, DET, DEA, DIA, DIHA, atrazine, terbuthylazine, simazine, acetochlor, and propazine | Diazinon (2A) | Diazinon | |
| Conejeros et al., 2024 (51) | Southern | Longitudinal | Seawater and sediment (Chiloé Island) | Cypermethrin, Deltamethrin | High. Cypermethrin and deltamethrin concentrations are higher than typical field levels but within laboratory experimental ranges | Not specified for additional agrochemical contaminants in the reviewed portion | No | No | |
| Cortés et al., 2020 (52) | South-Central | Longitudinal | Air | Chlorpyrifos | High. Compared with the Czech Republic, Canada, and Costa Rica, the chronic risk in children was determined using the methodology of the United States Environmental Protection Agency | Diazinon, atrazine, dimethoate, metolachlor, simazine, terbuthylazine, and tebuconazole | Diazinon (2A) | Diazinon | |
| Dutka et al., 1996 (53) | South-Central and South | Longitudinal | River water and sediment and untreated drinking water (freshwater systems) | Triazine (Temuco), atrazine, and benomyl (Rancagua). | High. Compared with European Commission regulations | Metolachlor and fecal bacteria | No | No | |
| Focardi et al., 1996 (54) | South-Central | Longitudinal | Fishes and birds of the Bío Bío River estuary (animal by-products and waste) | Lindane and PCBs | High. Lindane similar to levels in India and Russia, which are high, and PCBs above levels in fish from Japan, Australia, and the United States | DDT, HCHs, HCB, pentachlorobiphenyls, heptachlorobiphenyl, and hexachlorobiphenyls | Lindane (1), PCBs (1), DDT (2A), HCHs (2B), and HCB (2B) | No | |
| Giordano et al., 2011 (55) | South-Central | Longitudinal | River water (freshwater systems) | Lindane, cypermethrin, and fenvalerate | High. Compared with studies in the United Kingdom, Australia, and European Commission regulations | Malathion, diazinon, chlorpyrifos, and cyhalothrin | Lindane (1), diazinon (2A), and malathion (2A) | No | |
| Galbán-Malagón et al., 2023 (56) | Southern | Longitudinal | Air and surface seawater (Antarctic) | p,p′-DDT, o,p′-DDT, p,p′-DDE, o,p′-DDE | Low. Air, consistent with historical Antarctic studies).  Low. Water, compared to global and Antarctic standards | HCB and POPs | DDT (2A), HCB (2B), and DDT (2A) | No | |
| Gómez et al., 2023 (57) | Central | Longitudinal | Plastic debris in sandy beaches (Concepción Bay) | Polybrominated diphenyl ethers (PBDEs) (BDE, major contributor) | High. Levels of Σ10 PBDEs detected, predominantly BDE209 across all periods; concentrations peaked in summer | PCBs, OCs, DDTs, and HCHs | DDT (2A), HCHs (2B), PCBs (1) | No | |
| Gouin et al., 2019 (58) | Northern | Longitudinal | River sediments | Diethanolamine | Low. Compared with Europe and the United States | Atrazine, carbofuran, permethrin, diethanolamine | Diethanolamine (2B) | Diethanolamine | |
| Grimalt et al., 2004 (59) | Northern, South, and Southern | Longitudinal | Mosses in lake basins (animal by-products and waste) | HCHs | Low. Compared with United State, Australia, Canada, and Europe | DDT, and HCB | HCHs (2B), DDT (2A), and HCB (2B) | No | |
| Harner et al., 2006 (60) | Mountains of Chile and other countries | Longitudinal | Air | Endosulfan | Low. Compared with North Africa, India, and North America | HCHs, chlordane, and endosulfan | HCHs (2B), and chlordane (2B) | No | |
| Henríquez et al., 2006 (61) | South-Central | Cross-sectional | Soil | Aldrin | High. Compared with regulations of the European Commission, United States Environmental Protection Agency, and Agricultural and Livestock Service (SAG) | Dieldrin, DDT, DDE, and hydrocarbons | Aldrin/dieldrin (2A), and DDT (2A) | No | |
| Herrera-Muñoz et al., 2024 (62) | Metropolitan | Longitudinal | Surface waters of the Mapocho River impacted by treated effluent discharge | Atrazine, diuron, and terbuthylazine | Low. Concentrations of atrazine, diuron, and terbuthylazine were moderate compared to other Chilean river studies | Carbendazim, Imidacloprid, Simazine, Propamocarb, Metalaxyl, Acetamiprid, Tebuconazole, DEA, 2-Hydroxyterbuthylazine, Thiabendazole, Thiamethoxam. Pharmaceuticals (e.g., macrolides, quinolones, NSAIDs), drugs of abuse (e.g., cocaine metabolites), and ARGs | No | No | |
| Inostroza et al., 2024 (63) | Central | Longitudinal | Surface waters of the Aconcagua River basin | Diazinon, chlorpyrifos, and terbuthylazine | High. Diazinon and chlorpyrifos exceeded acceptable thresholds, posing significant risks to aquatic life. Terbuthylazine was moderate in concentration | Methomyl, clarithromycin, trenbolone, galaxolide, and benzothiazole | Diazinon (2A) | Diazinon | |
| Jara-Carrasco et al., 2017 (64) | Southern | Cross-sectional | Penguin feces (animal by-products and waste) | DDT | Low. Compared with Northern Hemisphere and European Commission | PCBs, endrin, and heptachlor | DDT (2A), PCBs (1), and heptachlor (2B) | No | |
| Llanos et al., 2022 (65) | South-Central | Longitudinal | Air | HCHs | Low. Compared with Mexico, Nepal, and South Africa | DDT, DDD, and PCBs | HCHs (2B), DDT (2A), and PCBs (1) | No | |
| Lohmann et al., 2023 (66) | South-Central and South | Longitudinal | Surface waters (Concepctión Bay and Llanquihue Lake) | PCBs, DDT | High. Elevated concentrations near Concepción Bay indicate local sources. | HCHs, HCB, dieldrin, endosulfan, and chlordane | PCBs (1), DDT (2A), HCB (2B), HCHs (2B), chlordane (2B) and dieldrin (2A) | No | |
| Luarte et al., 2022 (67) | South | Longitudinal | Air and water (freshwater systems) | HCB | Low. Compared with Russia, Canada, United States, and China | PCBs and HCB | HCB (2B) and PCBs (1) | No | |
| Luarte et al., 2024 (68) | Southern | Longitudinal | Air and seawater (Fildes Bay, King George Island, Antarctica) | 4,4′-DDT, 4,4′-DDE and HCB | High. HCB was detected in both air and seawater, highlighting its persistence as a global pollutant  Low. 4,4′-DDT and 4,4′-DDE detected at low levels, consistent with historical Antarctic data and legacy contamination | PCBs, α-HCH, and γ-HCH | HCB (2B) and PCBs (1) |  | |
| Mejías et al., 2021 (69) | South | Longitudinal | Honey and beeswax from beehives on farms (animal by-products and waste) | Fenhexamid (wax) | Low. Compared with the European Union | Phenols and antioxidants | No | No | |
| Mejías et al., 2019 (70) | Central and South-Central | Longitudinal | Honey bees on farms with hives (animal by-products and waste) | Thiamethoxam | High. Compared with the European Union | Thiacloprid, acetamiprid, imidacloprid, phenols, and antioxidants | No | No | |
| Montory et al., 2017 (71) | South-Central | Longitudinal | River water (freshwater systems) | Endosulfan | Low. Compared with the European Union, India, Brazil, and EPA | Lindane and DDT | Lindane (1) and DDT (2A) | No | |
| Montory et al., 2020 (72) | Southern | Cross-sectional | Chinook salmon (animal by-products and waste) | PCBs | High. Compared to EPA standards in adult fish | PBDEs, HCHs, DDT, and HCB | PCBs (1), HCHs (2B), DDT (2A), and HCB (2B) | No | |
| Muñoz-Arango et al., 2023 (73) | Northern, Central, Metropolitan, South-Central, and South | Longitudinal | Drinking water (freshwater systems) | Perchlorates and chlorates | High. Compared to EPA standards | Chlorate, chlorite, chloride, nitrate, nitrite, sulfate, phosphate, bromide, and fluoride | No | No | |
| Muñoz-Arnanz et al., 2019 (74) | South | Longitudinal | Blue whale blubber (animal by-products and waste) | PCBs | Low. Compared with Canada, Argentina, and Australia | HCB, DDT, and PBDE | PCBs (1) | No | |
| Muñoz-Quezada et al., 2014 (75) | South-Central | Cross-sectional | Soil | Linuron, sulfur, and cypermethrin (soil) | High. Only cypermethrin compared to European Union standards | Linuron, cypermethrin, and DDE-pp | No | No | |
| Palma et al., 2004 (76) | South | Longitudinal | River water (freshwater systems) | 2,4-D, simazine, hexazinone, and carbendazim | High. Compared to the Chilean and European Union standards (simazine and 2,4-D) | Pyroclam | 2,4-D (2B) | 2,4-D | |
| Palma-Fleming et al., 2000 (77) | South | Longitudinal | Solid waste and sediments from the municipal landfill and sediments from the Futa River and Calle Calle River | Heptachlor and endosulfan | High. Compared to EPA standards | α-BHC, β-BHC, 4,4’-DDE, dieldrin, endrin aldehyde, endosulfan sulfate and heptachlor, and high levels of Fe, Zn, Cu, Cr, Ni, and Pb | Heptachlor (2B) and dieldrin (2A) | No | |
| Palma-Fleming et al., 2008 (78) | Central and South | Longitudinal | Mussel Perumytilus purpuratus (Lamarck) (animal by-products and waste) | PCBs | High. Compared to EPA standards | PAHs | PCBs (1) | No | |
| Placencia et al., 2018a (79) | South | Longitudinal | River water and sediment | HCHs, DDE, and endosulfan | High. Compared to Asia | DDT and DDD | HCHs (2B) and DDT (2A) | No | |
| Placencia et al., 2018b (80) | South | Longitudinal | Fjord sediments (marine) | Deltamethrin | High. Compared with United States, Norway, and Netherlands | Diflubenzuron and organic carbon | No | No | |
| Pozo et al., 2004 (81) | South-Central | Longitudinal | Air | Endosulfan | High. Compared to the Canadian Arctic and Senga Bay, Africa (endosulfan in Chungará Lake and Laja Lake) | HCHs, PCBs, TC, CC, and toxaphene | HCHs (2B), PCBs (1), and chlordane (2B) | No | |
| Pozo et al., 2012 (82) | South-Central | Longitudinal | Air | PCBs | High. As required by the Stockholm Convention | PAHs, DDT, DDE, HC, chlordane, heptachlor, dieldrin, endosulfan, and PBDEs | PCBs (1), DDT (2A), chlordane (2B), heptachlor (2B), and dieldrin (2A) | No | |
| Pozo et al., 2014 (83) | South-Central | Longitudinal | Sediments | PCBs | High. Compared with Argentina, India, and China. As required by the Stockholm Convention. | HCB and Ocs | PCBs (1) and HCB (2B) | No | |
| Pozo et al., 2016  (84) | South | Longitudinal | Air | Chlorpyrifos | High. Compared with Czech Republic, Ireland, France, Canada, and Costa Rica | N/A | No | No | |
| Pozo et al., 2017 (85) | Metropolitan, South-Central, and South | Longitudinal | Air | PCBs and PBDE | Low. Compared with Canada and African urban areas | HCHs and DDT | PCBs (1), DDT (2A), and HCHs (2B) | No | |
| Pozo et al., 2022a (86) | South-Central | Longitudinal | Air, water, sediment and soil | PCBs | Low. Compared wth India, Spain, Argentina, and South Korea | PAHs, PBDEs, and DDT | PCBs (1) and DDT (2A) | No | |
| Pozo et al., 2022b (87) | South-Central | Longitudinal | Air and water | PBDE | Low. Compared with Europe and Canada | PAHs, PCBs, and DDT | PCBs (1) and DDT (2A) | No | |
| Toro et al., 2004 (88) | South-Central and South | Longitudinal | Giant mussel Choromytilus Chorus (animal by-products and waste) | OCs | Low. Compared with Canada, Africa, China, Spain, and United States | HCHs, aldrin, endrin aldehyde, heptachlor epoxide, HCB, DDE, DDT, and PAHs | HCHs (2B), aldrin (2A), heptachlor (2B), HCB (2B), and DDT (2A) | No | |
| Schofer et al., 2024 (89) | South-Central | Cross-sectional | Agricultural soils (vineyards and orchards) | Cu-based pesticides | High. Cu-bases pesticides levels ranged from 23 to 566 mg/kg, exceeding the EC50 for earthworm avoidance (240 mg/kg) and global soil quality guidelines for ecological health (≤100 mg/kg) | Zn, As, Pb | As (1) | As | |
| Soriano et al., 2024 (90) | Central, Metropolitan | Longitudinal | Surface water and sediment (River Maipo and  Aconcagua basins) | Imazalil, azoxystrobin acid, Cyromazine | High. Imazalil exceeds typical levels in Europe and South America. Other pesticides are within acceptable ranges globally | PAHs, PFAs, and PCPHP | No | No | |
| Tovar et al., 2023 (91) | Northern | Cross-sectional study | Coastal sediments (Atacama: Chanaral, Copiapó, and Huasco) | NPs | High. NPs concentrations were significantly higher than global studies in Brazil and the U.S. | Chlorpyrifos, pyrene, benzo[a]pyrene, chrysene, naphthalene, phenanthrene, DDE, permethrin, and phenothrin | Benzo[a]pyrene (1), benz[a]anthracene (2B), and naphthalene (2B) | No | |
| Tucca et al., 2017 (92) | South | Longitudinal | Sediment salmon cages 4 farming centers (animal by-products and waste) | Cypermethrin | High. Compared with Argentina, India, European Union, and EPA-United States | Emamectin benzoate, diflubenzuron, and teflubenzuron | No | No | |
| Vergara et al., 2019 (93) | Southern | Cross-sectional | Penguin feces and fat (animal by-products and waste) | DDT, HCHs and methoxychlor | High. Compared with EPA United States and China. | HC, HCB, aldrin, dieldrin, chlordane, endosulfan, Methoxychlor, DDE, and DDT | DDT (2A), HCHs (2B), HCB (2B), aldrin/dieldrin (2A), and chlordane (2B) | No | |
| Vorkamp et al., 2010 (95) | South | Longitudinal | Bivalves (animal by-products and waste) | DDT | Low. Compared with Australia and United States | PCBs, HCHs, and PAHs | DDT (2A) and PCBs (1) | No | |

**Abbreviation:** 137Cs = Cesium-137; 2,4-D = 2,4-dichlorophenoxyacetic acid; 4,4'-DDE = Dichlorodiphenyldichloroethylene; α-BHC = Alpha-Benzenehexachloride; β-BHC = Beta-Benzenehexachloride; ARGs = Antibiotic Resistance Genes; BDE = Brominated Diphenyl Ethers; BDEE209 = Brominated Diphenyl Ether 209; BHC = Benzenehexachloride; CC = Choline Chloride; Cr = Chromium; Cu = Copper; DDE = Dichlorodiphenyldichloroethylene; DDE-pp = Dichlorodiphenyldichloroethylene, Para-Para Isomer; DDD = Dichlorodiphenyldichloroethane; DDDE = Dichlorodiphenyldichloroethane; DDT = Dichlorodiphenyltrichloroethane; DEA = Deethylatrazine; DET = Desethylterbuthylazine; DIA = Deisopropylatrazine; DIHA = Deisopropylhydroxyatrazine; Drins = Aldrin, dieldrin, endrin, and isodrin; Fe = Iron; HA = Atrazine-2-hydroxy; HCB = Hexachlorobenzene; HC = Hydrocarbons; HCHs = Hexachlorocyclohexanes; Ni = Nickel; N/A = Not Applicable; NPs = Nonylphenols; NSAIDs = Non-Steroidal Anti-Inflammatory Drugs; OCs = Organochlorides; PAHs = Polycyclic Aromatic Hydrocarbons; PBDEs = Polybrominated Diphenyl Ethers; PCBs = Polychlorinated Biphenyls; PCPHP = Personal Care Products and Household Products; PFAs = Per- and Polyfluoroalkyl Substances; POPs = Persistent Organic Pollutants; Pb = Lead; TC = Total Chlorine; Zn = Zinc.

***Clarification notes:** 137Cs, ARGs, As, BDE, BDEE209, CC, Cr, Cu, DET, DIA, DIHA, Fe, HA, HC, Ni, NPS, NSAIDs, PAHs, PBDEs, PCBs, PCPHP, PFAs, POPs, Pb, TC, Zn are not pesticides, but persistent organic pollutants that enter the environment through similar pathways. Their inclusion in the table aims to provide a more comprehensive view of toxic chemical exposure in agricultural systems, as they can interact with pesticides and increase risks to human health and the environment.
